# A Model for Chagas Disease with Oral and Congenital Transmission

**DOI:** 10.1371/journal.pone.0067267

**Published:** 2013-06-28

**Authors:** Daniel J. Coffield, Anna Maria Spagnuolo, Meir Shillor, Ensela Mema, Bruce Pell, Amanda Pruzinsky, Alexandra Zetye

**Affiliations:** 1 Mathematics Department, University of Michigan-Flint, Flint, Michigan, United States of America; 2 Department of Mathematics and Statistics, Oakland University, Rochester, Michigan, United States of America; 3 Department of Mathematical Sciences, New Jerseys Science & Technology University, University Heights, Newark, New Jersey, United States of America; 4 School of Mathematical & Statistical Sciences, Arizona State University, Tempe, Arizona, United States of America; 5 Chesapeake Research Consortium, U.S. EPA Chesapeake Bay Program Office, Annapolis, Maryland, United States of America; Albert Einstein College of Medicine, United States of America

## Abstract

This work presents a new mathematical model for the domestic transmission of Chagas disease, a parasitic disease affecting humans and other mammals throughout Central and South America. The model takes into account congenital transmission in both humans and domestic mammals as well as oral transmission in domestic mammals. The model has time-dependent coefficients to account for seasonality and consists of four nonlinear differential equations, one of which has a delay, for the populations of vectors, infected vectors, infected humans, and infected mammals in the domestic setting. Computer simulations show that congenital transmission has a modest effect on infection while oral transmission in domestic mammals substantially contributes to the spread of the disease. In particular, oral transmission provides an alternative to vector biting as an infection route for the domestic mammals, who are key to the infection cycle. This may lead to high infection rates in domestic mammals even when the vectors have a low preference for biting them, and ultimately results in high infection levels in humans.

## Introduction

Chagas disease is caused by infection with the parasite *Trypanosoma cruzi* and is a major source of suffering throughout Latin America. The disease leads to organ deformity and early death in about one third of the 8–10 million individuals infected, [1], [2]. Vector transmission by reduviids is largely responsible for the spread of *T. cruzi*, with some particular species specialized in domestic infection cycles. Other modes of transmission include blood tranfusions, organ transplants, oral transmission, and congenital transmission [3]–[6].

Although various drugs are under development and testing, current control of the transmission of Chagas disease remains largely based on vector control and blood-bank screening [7]–[10]. In particular, the Southern Cone Initiative was implemented in the 1990s with the goal of interrupting the transmission of Chagas disease in South American countries through insecticide spraying and blood screening [10]–[12]. This program has led to a dramatic decrease in transmission in several countries in South America, with some regions now reporting a considerable drop in infections from *Triatoma infestans*, the primary vector, and transmission virtually at zero [13], [14]. Additional control measures are treatment for acute Chagas disease and for congenital transmission cases [15].

While insecticide spraying for Chagas vectors has led to a significant decrease in new infections, improved housing, better drugs, and an effective vaccine are needed [16]. In particular, *T. cruzi* infection is likely to remain endemic in sylvatic hosts despite spraying efforts and neither insect control nor current drug treatment is optimal for this disease because of the long life span of infected human hosts, triatomine insecticide resistance, and the ease with which protozoans develop drug resistance [17]–[20].

Mathematical models for studying Chagas disease dynamics with seasonal insecticide spraying were presented in [21], [22]. In this work, we enhance the model in [21], adding the effects of congenital transmission in infected humans and infected dogs and excluding spraying. We also account for oral transmission by allowing the domestic mammals to consume the vectors, as observed experimentally in [23]. The predation term involves a density-dependent consumption rate in the form of a Holling Type II response, similar to that in [4]. There are other likely routes of oral transmission in domestic mammals such as ingesting feces-contaminated food and water or licking feces-contaminated fur. Though the vector consumption is derived on the basis of the animals preying on the vectors, the consumption term can still in some sense account for these other modes since the consumption is dependent on the vector density.

The primary aim of this work is to investigate the significance of the following modes of disease transmission relative to vector biting: 1.) oral transmission due to predation, and 2.) congenital transmission. In particular, we want to know if these transmission modes play a significant role in human infection and have implications for disease control. To this end, we study the additional transmission modes as enhancements to the model in [21] for comparison.

The model we present here consists of four nonlinear differential equations that describe the domestic transmission of the disease by predicting the total number of vectors, infected vectors, infected humans, and infected domestic mammals. In [24], a mathematical model for a small population (one household) was described, whereas the model in this work considers a large population (one village). We note that models were recently used in [25], [26] to study control issues of the non-domiciliated Chagas disease vectors in the Yucatan Peninsula, Mexico. Those models use difference equations while our model is a classical nonlinear dynamical system. The effectiveness of different control measures was recently studied in [27] using a dynamical system model. Additional modeling and field results can be found in [28]–[30].

The Methods section gives a detailed description of the model and its parameters. In Results, the model is used to produce simulations of the populations in a hypothetical rural village over thirty years using a baseline parameter set, provided in Table 1. Additional simulations are performed to investigate the model's sensitivity to various parameters. The Results section also compares simulations of the model presented in this work with the model in [21], which does not consider oral transmission in dogs nor congenital transmission. We conclude with the Discussion.

**Table 1:** The model parameters and the baseline simulation values.

Parameter	Definition	Baseline Simulation Value	Source
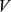	Total number of vectors (vectors/village)	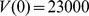	
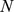	Total number of humans (humans/village)	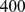	This study
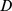	Total number of domestic dogs (dogs/village)	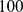	This study
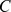	Total number of chickens (chickens/village)	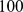	This study
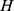	Total number of houses (houses/village)	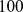	This study
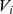	Infected domestic triatomids (vectors/village)	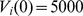	This study
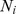	Number of infected humans (humans/village)	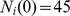	This study
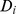	Number of infected dogs (dogs/village)	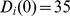	This study
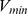	Min. number of vectors (vectors/village)	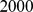	This study
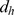	Egg hatching rate (1/day)	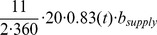	Figure 1, [37], [38]
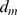	Death rate of vectors (1/day)	Seasonal piecewise linear	Estimation from [38], Figure 1
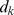	Death rate of vectors (above 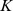 ) (1/day)	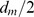	This study
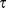	Delay (days)	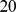	[38]
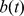	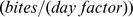 Biting rate	Seasonal piecewise linear	Estimation from [38], [39], Figure 1 in [21]
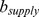	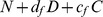	Seasonal piecewise linear	This study
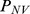	Human to vector infection probability (per bite)	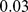	[24]
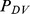	Dog to vector infection probability (per bite)	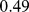	[24]
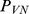	Vector to human infection probability (per bite)	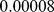	This study, value unknown
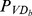	Vector to dog infection probability (per bite)	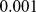	Estimate from [24]
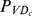	Vector to dog infection probability via oral consumption	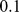	[40]
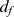	Human factor of one dog	2.45	[34]
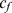	Human factor of one chicken	0.35	[34]
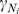	Mortality rate of infected humans (1/day)	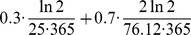	Estimate from [41], [42]
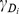	Mortality rate of infected dogs (1/day)	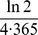	Estimate 8 years
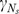	Mortality rate of susceptible humans (1/day)	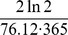	Estimate from [41], [42]
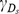	Mortality rate of susceptible dogs (1/day)	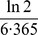	Estimate 12 years
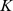	Carrying Capacity per village	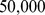	This study
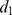	First day of fall (day of year)	0	March 20
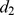	First day of winter (day of year)	91.25	June 21
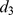	First day of spring (day of year)	182.5	September 22
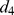	First day of summer (day of year)	273.75	December 21
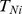	Congenital transmission probability for infected humans	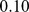	[5], [15], [40]
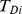	Congenital transmission probability for infected dogs	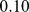	[5], [15], [40]
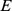	Max. number of vectors eaten by a dog per day	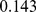	This study
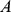	Number of vectors when vector consumption is E/2	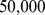	This study

## Methods

In this section, we present a new model for Chagas disease dynamics in a village. Information on pertinent biological processes can be found in [24]. The model represents the overall dynamics of the populations of vectors, infected vectors, infected humans, and infected domestic animals (mammals only) – referred to as 'dogs' in our model. In a rural village, there are also non-mammals that act as sources of blood meals but that are not hosts for *T. cruzi* (i.e., cannot become infected). They will be referred to as 'chickens' in our model.

We consider a relatively large representative rural village in South America so that a differential equations model is appropriate. Let the number of humans in the village be 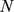, the number of domestic mammals (dogs) be 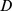, and the number of domestic non-mammals (chickens) be 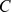. These are taken as constants for the sake of simplicity, since we consider a modest time period of 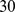 years. We denote by 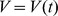 the number of carrier insects living in the houses in the village at time 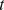, the number of infected insects by 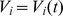, the number of infected humans by 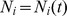, and the number of infected dogs by 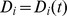. Each non-infected population, excluding the chickens, is assumed to be susceptible.

We now describe the rate of change of each population in the village per day. The growth rate of the vectors depends on the successful hatching of eggs. As in [22], the egg hatching rate, 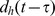, is delayed due to the gestation time of 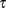 days. The hatching rate is a product of the following terms: the ratio of adult female vectors to total vectors; the number of eggs laid by an adult female per bite; the successful hatching rate of eggs after 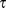 days; the total blood meal supply (in human factors), 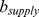; and the delayed seasonal biting rate 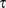 days prior to hatching, 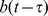, in units of bites per vector per day per human factor (Table 1). By extracting seasonal data from Castanera et al. [38], we obtain the following values: the ratio of adults to the entire population of triatomines is approximately 11/365 (so we take half that number to be adult females), the fraction of eggs that survive is 0.83, and the number of (eggs/bite)/(fed female) is 20 [37], [38]. So, the form of 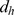 is
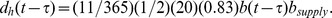



Note that we are assuming 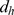 follows the seasonality of 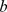.

Following [24], we use 'human factors' as the unit for the total blood supply in the following way: each human represents one human factor, each mammal 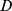 represents 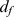 human factors, and each non-mammal 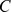 represents 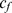 human factors; then, the total blood supply is given by 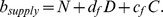 We use the standard notation 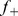 and 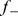 to denote the positive and negative parts, respectively, of a function 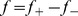. Vector growth is then modeled by the following delay logistic term
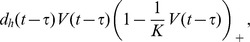
as in [21], where 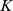 is the carrying capacity of vectors in the village houses. The term 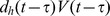 is the rate at which vectors hatch at time 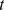 from eggs laid at time 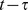. The expression 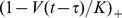 represents the fraction of the food supply that was available to the female vectors at time 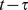. This assumes that if the vector population at time 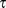 days prior to the current time 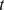 was above the carrying capacity, then no eggs are laid. The death rate of the vector population depends on the following three factors: natural mortality, death due to overcrowding or growth beyond the carrying capacity, and death due to being eaten by the dogs. The natural death rate coefficient of triatomines is 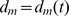 and the coefficient of the death rate of vectors above the carrying capacity is 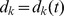. These rates are assumed to be periodic functions with a period of one year and are included by adding the following term to the vector growth equation:



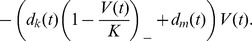



We now consider the death rate of vectors due to consumption by dogs. We use a Holling Type II functional response for the consumption term, defining the per dog consumption rate as
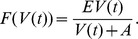



Here, 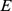 is the maximum number of vectors consumed by one dog per day and 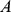 is the vector number at which dogs consume at the rate of 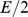 vectors per day. This term is similar to one used in [4] where vector consumption by wild animals was considered. Thus, the rate of change in the total vector population within the village is
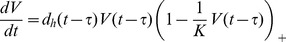


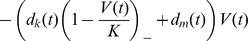


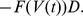



Next, we consider the growth rates of the infected populations 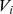, 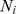, and 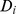. We denote by 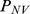 and 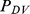 the probabilities of a vector becoming infected by biting an infected human or an infected dog, respectively. Also, the number of bites per vector per day is given by 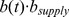, where 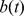 is the same seasonal biting term used to define 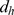. Since the proportions of those bites that occur on infected humans and infected dogs are 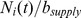 and 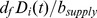, respectively, the growth rate of infected vectors is
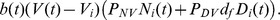



We assume that the natural death rate of the infected vectors is also 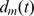, i.e., carrying the parasites does not affect their life span. Similarly, the death rate due to growth beyond the carrying capacity is 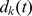.

The death rate of the infected vectors due to predation by dogs is
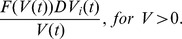



Collecting the terms above, we get the following rate of change for the infected vectors:
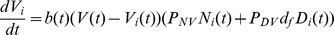


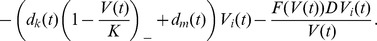



We turn now to the infected humans. When bitten by an infected vector, a susceptible human becomes infected with probability 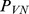. As before, each vector is biting at a rate of 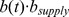 bites per day, and 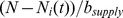 is the fraction of bites that are on susceptible humans. Thus, the growth rate of infected humans is given by 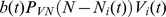. The natural mortality rates for infected humans and susceptible human are denoted by 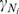 and 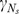, respectively. We assume that human reproduction is independent of infection status, since infected humans typically live beyond reproductive ages. Therefore, the assumption that 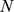 is constant implies that the birth rate for all humans is
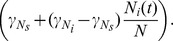



Here, the birth rate depends on 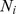, which is a consequence of the two preceding assumptions. However, we note that 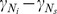 is small so that 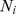 does not vary enough for this dependence to meaningfully affect the model. Furthermore, we investigated multiple other assumptions for the human birth rate, including scenarios for different birth rates of infected humans versus uninfected humans, and the simulation results were virtually indistinguishable in all cases.

We define 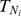 to be the ratio, amongst babies born to infected mothers, of infected babies to the total number of babies. Therefore, the rate of change of infected humans is
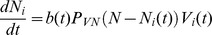


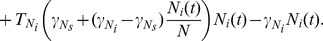



Under the assumption that both infected and uninfected dogs reproduce at the same rate, the rate of change of infected dogs is similar to that of infected humans, except that we must account for infection caused by the consumption of infected vectors. To this end, let 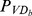 be the probability that an uninfected dog becomes infected when bitten by an infected vector, let 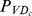 be the probability that an uninfected dog becomes infected after eating an infected vector, and note that 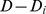 be the susceptible dog population. We also use 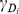 and 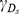 to denote the natural mortality rates for infected dogs and susceptible dogs, respectively. Then, the rate of change of infected dogs is
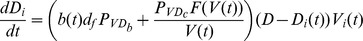


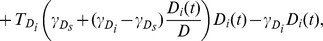
where 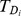 is the probability that an infected dog passes the infection to its offspring congenitally, and the congenital transmission term is similar to the term in the infected humans equation because 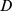 is constant.

To complete the model we prescribe the initial values of the respective populations: 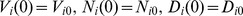, together with.



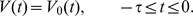
.

These equations and conditions form a mathematical model for the domestic dynamics of Chagas disease with oral and congenital transmission:
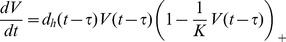


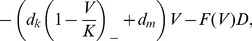
(1)

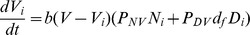


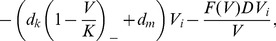
(2)

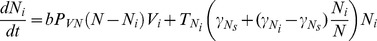


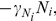
(3)

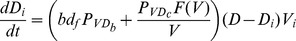


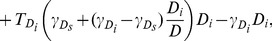
(4)

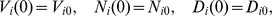


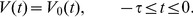
(5)


The coefficient functions 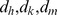, and 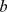 are one-year periodic since they are seasonally dependent. Also, it is natural to assume that 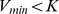. Note that the delay differential equation (1) for the total vector population is not coupled to the other equations, so it can be solved independently.

## Results

In this section, we study the effects of the congenital and oral transmission terms, various model parameters, and compare the current model with the one in [21]. The Adams-Bashforth Fourth-Order Method was implemented in gfortran [31] and verified using Wolfram Mathematica [32]. The figures were generated with Wolfram Mathematica [32].

### Baseline Case

We first compare the simulation results in a baseline case, similar to the one described in [21], with 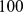 houses, 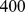 humans, 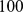 dogs, and 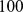 chickens in a respresentative village. We use a similar parameter set to define our baseline case with the only difference being a new value of 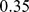 for 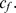 See Vector Biting Preference Studies for an explanation. We also add the parameters for congenital and oral transmission. See Table 1 for baseline parameters. The simulation timeframe is 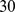 years. The values of 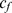 and 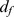 significantly affect the model, so we investigate them further in the next section.

**Figure 1:**
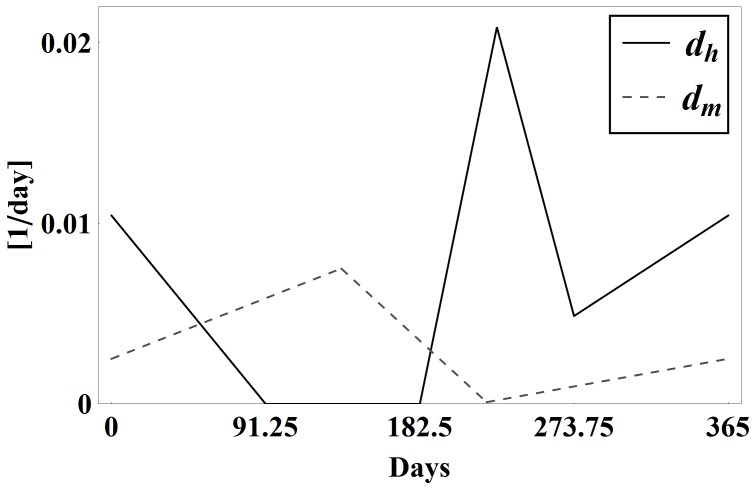
Vector growth and mortality coefficients. The vector growth rate coefficient 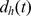 in the baseline case and the vector mortality rate coefficient 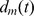 over one year.

The probabilities for congenital transmission are taken from data in [5], [15], [40]. Since data for predation rates of domestic mammals is not currently available, we estimate the maximum value of the per dog vector consumption rate to be about 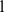 vector per week. This estimate is within the range of values estimated for wild mammals in [40]. For the probability of dog infection via oral consumption of an infected vector, we use the value found in [40], which is a weighted average of values found from experiments with raccoons and opossums. An appropriate value of the parameter 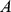 is not known. We chose it to be 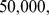 meaning that when a village house has 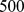 vectors in it, then dogs consume vectors at a rate of 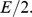 We note that although the model is sensitive to the total number of vectors, it is not sensitive to the value of 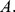 Also, Figure 1 contains the graph of the seasonally dependent values of 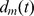 and 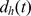. See Table 1 and the references therein for the data used to estimate these values and note that 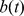 is proportional to 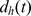.

**Figure 2:**
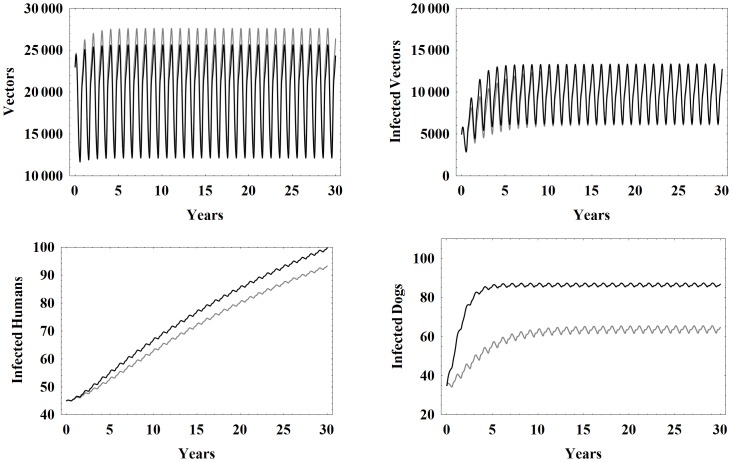
Simulation results comparing models. Simulation results of the model in this work (black) and the model without both oral and congenital transmission (gray), from [21], in the baseline case using the parameters in Table 1.

Figure 2 shows the seasonal oscillations of the total vectors, infected vectors, infected humans, and infected dogs for the models with and without congenital and oral transmission. Notice that the new model produces lower peaks for the total number of vectors whereas the infected human and infected dog populations take on larger values due to the new transmission modes. The infected vector population in the new model attains higher values early on, but within 10 years is nearly identical to the old model.

**Figure 3:**
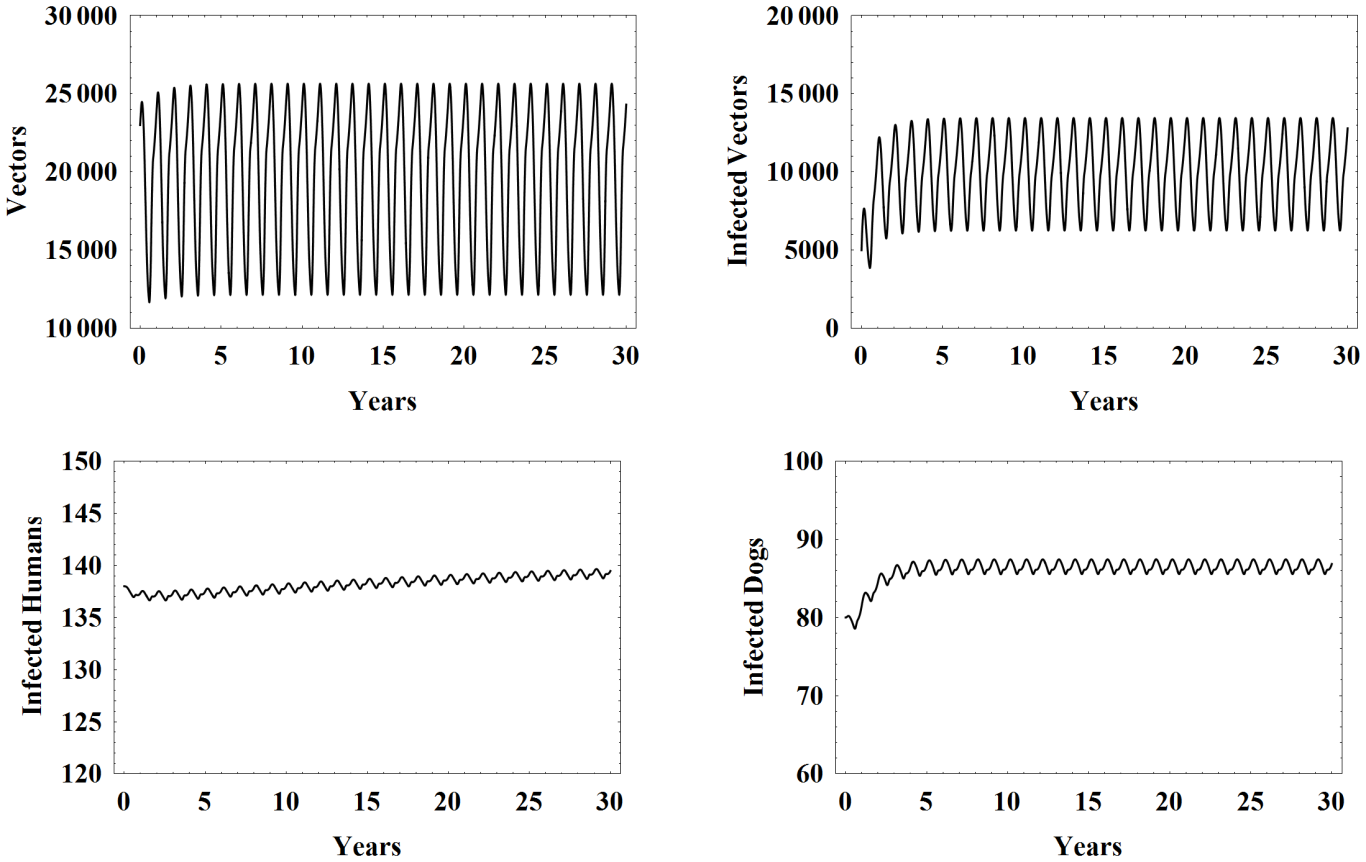
Higher initial conditions. Simulation results of the model with baseline parameters and higher initial conditions.

Figure 3 shows model simulations for the number of vectors, infected vectors, infected humans, and infected dogs over 30 years using the the baseline parameter set with higher initial conditions. We note that all of the populations largely stabilize around a central value and then seasonally oscillate around that value. This is consistent with the endemic nature of the disease in rural villages that do not engage in control measures. Note that the oscillations in the vector populations are much larger than those of the humans and domestic mammals, because of the shorter life span of vectors.

### Vector Biting Preference Studies

**Figure 4:**
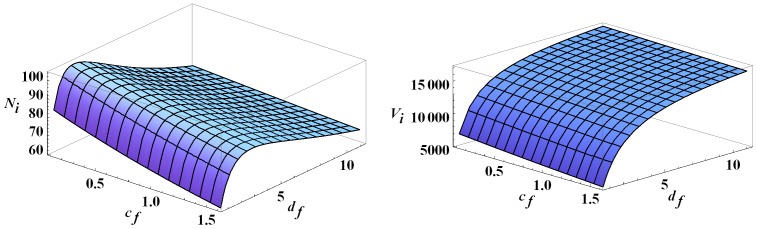
Infected human and vector populations at year 30. The infected human and infected vector populations at year 30 as functions of 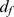 and 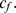 All other parameters are set to their baseline values.

Recently, experimental work in [33] suggests that 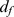 is about seven times 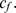 This strongly contradicts earlier estimates found in [34] and used in our previous work [21], [22]. We point out that this new study only compares vector preference between dogs and chickens, but does not consider vector preference for humans. Thus, vector preference between humans, dogs, and chickens remains unclear. Moreover, our work indicates that the preference factors substantially affect the dynamics of the infected populations. To address this, we perform simulations over various ranges of 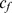 and 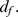 See Figure 4.

Note that for fixed 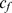 and increasing 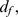 infection in humans increases and then decreases, attaining its peak for 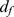 between 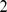 and 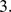 Although not shown, the number of infected dogs increases as 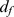 increases, as expected. This leads to a sharp increase in infected vectors for low 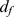 values before leveling off later on. See Figure 4. The initial increase in human infections is due to the steep increase in infected vectors which more than compensates for the increased vector preference for dogs. Because the number of infected vectors levels off for large values of 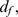 it follows that human infection decreases as the vectors primarily bite the dogs. Also, for each fixed 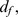 human infections decrease as 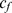 increases. This is expected since chickens are not infective and do not contribute to the infection cycle. Hence a higher 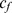 just diverts bites from dogs and humans.

In most of the following simulations, we allow 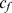 to vary and consistently choose 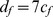 due to the relationship found in [33]. In simulations where 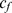 and 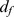 are fixed, we choose 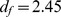 to stay consistent with baseline studies in previous work and correspondingly set 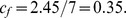


### Effects of Oral and Congenital Transmission

**Figure 5:**
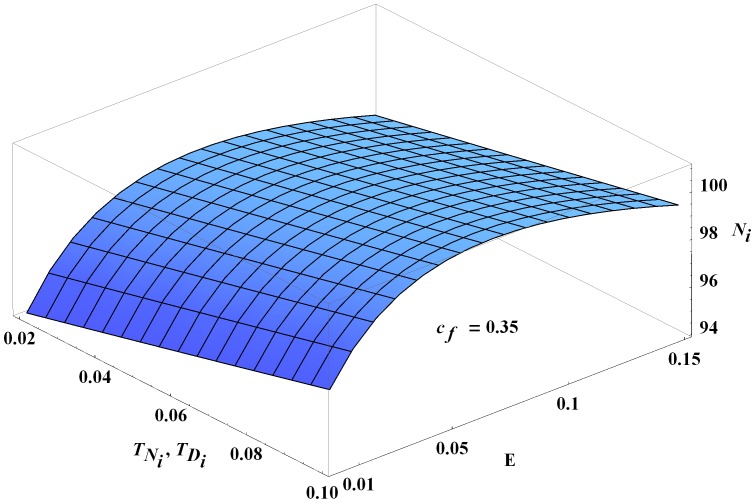
Infected humans at year 30 as a function of 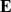, 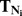, and 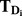. The number of infected humans at year 30 as a function of the vector consumption rate 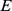 and congenital transmission probabilities 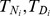 (where 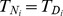). All other parameters are the baseline values.

As expected, congenital transmission increases overall infection in humans and dogs. As a result, the number of infected vectors also increases, but not significantly. Estimates of congenital transmission probabilities are readily available and generally in the range of 2–10% [5], [15]. For values in this range, the effects of congenital transmission are modest and close to linear as a function of transmission probabilities, Figure 5. We note that a recent article reported dramatically higher congenital transmission probabilities in mice (33–66%) [35]. Simulations of our model with congenital transmission probabilities up to 50% reveal a continued, near-linear effect of vertical transmission on infected humans.

**Figure 6:**
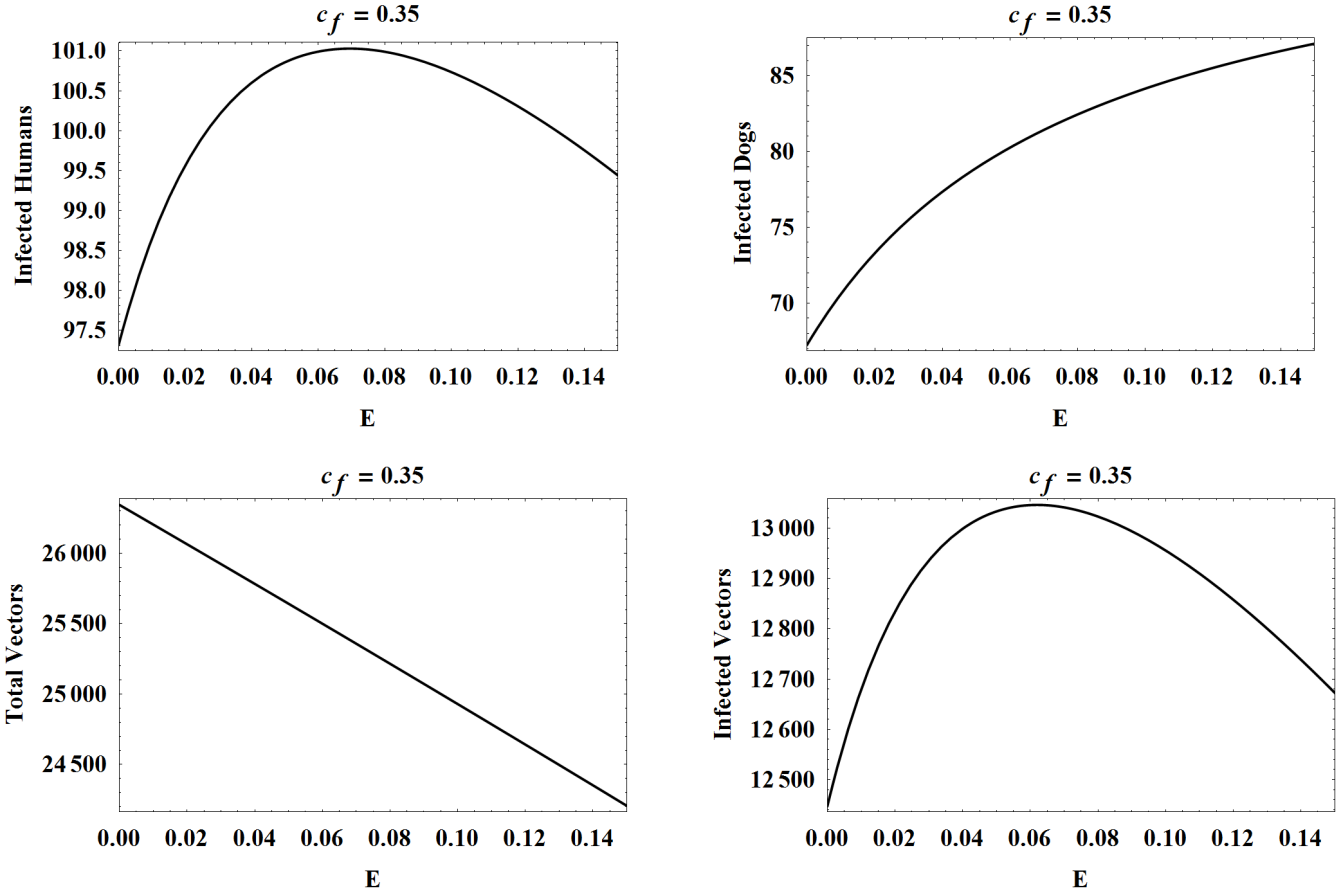
Populations at year 30 as functions of 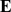. The number of infected humans, infected dogs, vectors, and infected vectors, all at year 30, as functions of 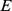. The other parameters are set to the baseline values.

The oral transmission is more complicated. In the case shown in Figure 5 with a baseline 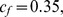 the number of infected humans at year 30 changes by at most 4 as 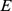 ranges from 0 to 0.15 with a peak attained at approximately 0.07. We note that initally a higher vector consumption rate results in more infections in both humans and dogs, as expected. However, as the consumption rate increases further, the number of human infections actually declines since there are fewer infected vectors feeding on humans, as depicted in Figure 6. Also, the total number of vectors steadily decreases, since more vectors are being consumed as 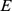 increases.

**Figure 7:**
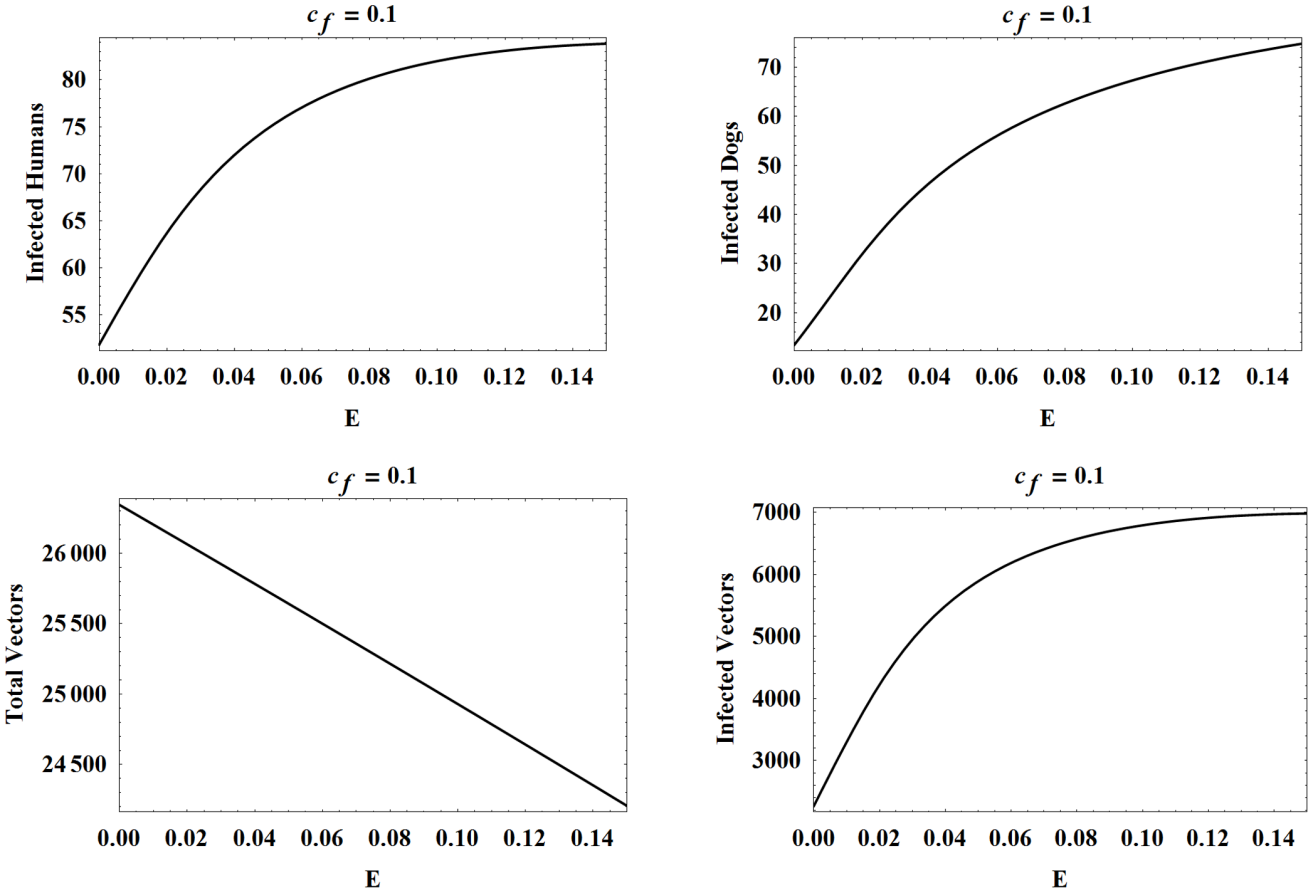
Populations at year 30 as functions of 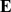 with 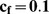. The number of infected humans, infected dogs, vectors, and infected vectors, all at year 30, as functions of 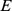. Here 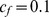 while all other parameters are set to the baseline values.

**Figure 8:**
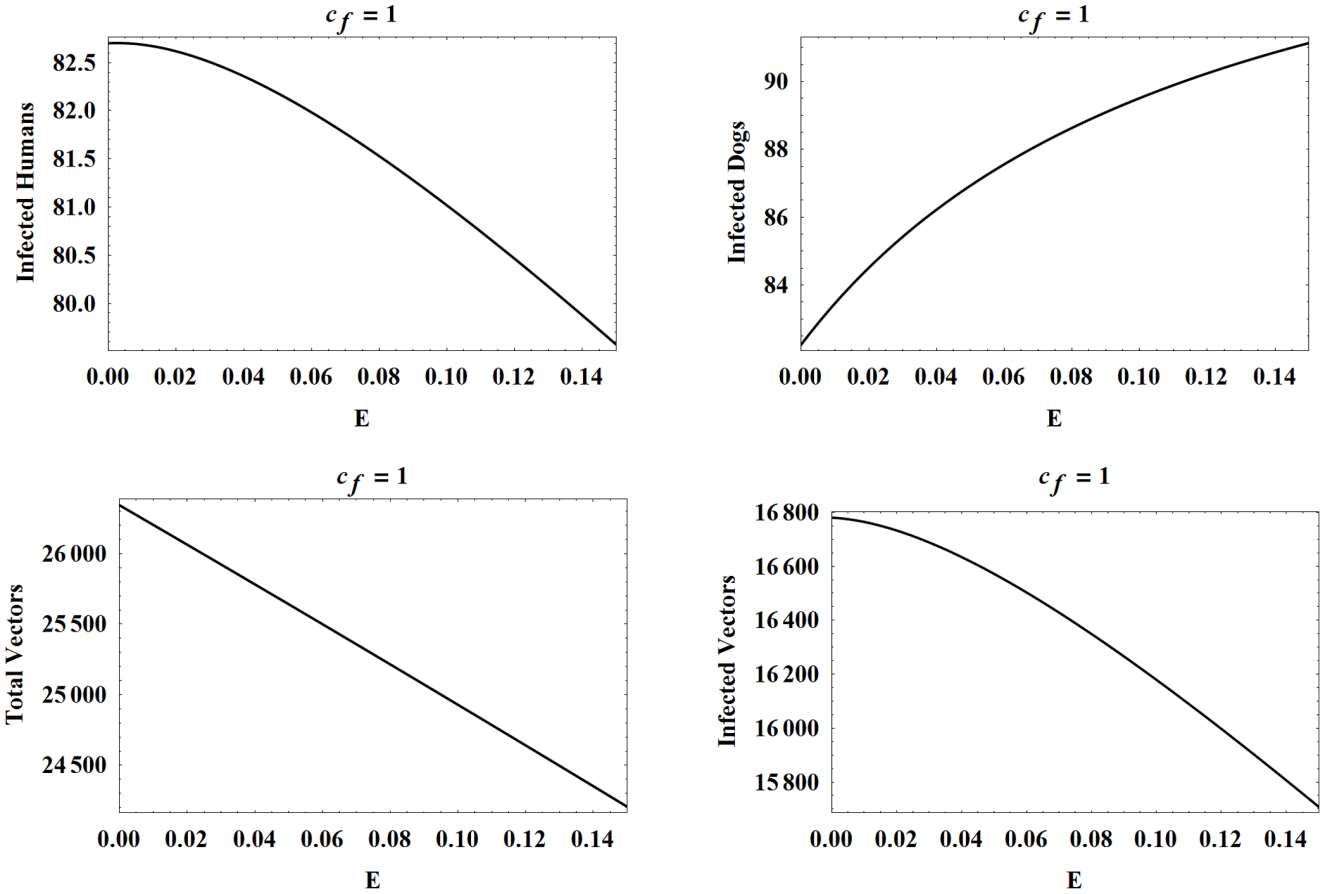
Populations at year 30 as functions of 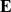 with 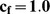. The number of infected humans, infected dogs, vectors, and infected vectors, all at year 30, as functions of 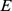. Here 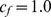 while all other parameters are set to the baseline values.

Next, we consider oral transmission with higher and lower values of 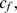 and, correspondingly, 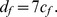 Figures 7 and 8 show the four populations as functions of 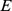 with all other parameters except 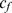 and 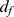 set to their baseline values. We point out that varying 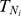 and 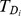 had the same effect on the populations as in Figure 5. In the case where 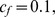 the effects of oral transmission are more dramatic with the number of infected humans at year 30 increasing by approximately 30 as 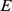 ranges from 0 to 0.15. In comparison, when 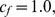 the number of infected humans starts at a significantly lower number and decreases. We observed similar trends with larger 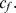


Since the vector preference numbers do not affect the biting rate of the vectors, it follows that the total number of vectors is the same for a fixed value of 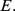 So, in each of Figures 6, 7, 8, the graphs for the total number of vectors are identical. However, all infected populations have dramatically different outcomes. More specifically, in all cases, the number of infected dogs increases as 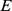 increases, because the dogs are eating more infected vectors. Also, dog infection increases with 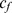 since 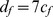.

Now, we consider the number of infected humans for the different values of 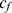. It is interesting to note that the curves for the number of infected humans and infected vectors have the same shape in each instance of 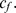 We observe that for 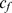 roughly less than 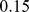 (and therefore, 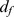 roughly less than 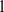), the curves for infected humans and infected vectors are increasing with 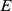 in 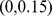. However, for 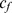 roughly in the interval 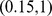, the curves initially increase before decreasing with the peak moving to the left for higher values of 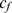. Also, it appears that the peak of each of the 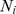 curves trails closely behind the peak of the corresponding 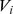 curves since vector infection drives human infection.

We now explain the decline after the peak for infected vectors, and correspondingly, for infected humans. Recall that 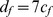, so a higher 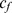 value means a stronger vector preference for dogs and a resulting higher number of infected vectors. However, since the total number of vectors is independent of 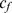, the ratio 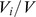 increases as 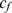 increases. Also, 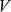 is a decreasing function of 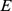, making 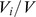 even higher for large 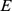. Thus, more of the vectors removed through dog predation are infected vectors for higher 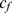 and 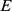 values. Initially as 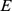 increases, more dogs become infected which in turn leads to a higher number of infected vectors. But, as 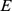 increases further, more infected vectors are being eliminated through vector consumption than are being added through new infections.

Finally, for 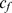 greater than about 1, the number of infected humans and infected vectors declines as 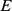 increases from 0 to 0.15. As in the latter part of the previous case, the high vector preference for dogs leads to a large infected vector population and a high 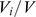 ratio. The predation on vectors removes such a high proportion of infected vectors in this case that the number of infected vectors strictly decreases as a 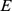 increases. Dog infection still increases with 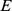, leading to new vector infections, but not enough to overcome those being removed.

**Figure 9:**
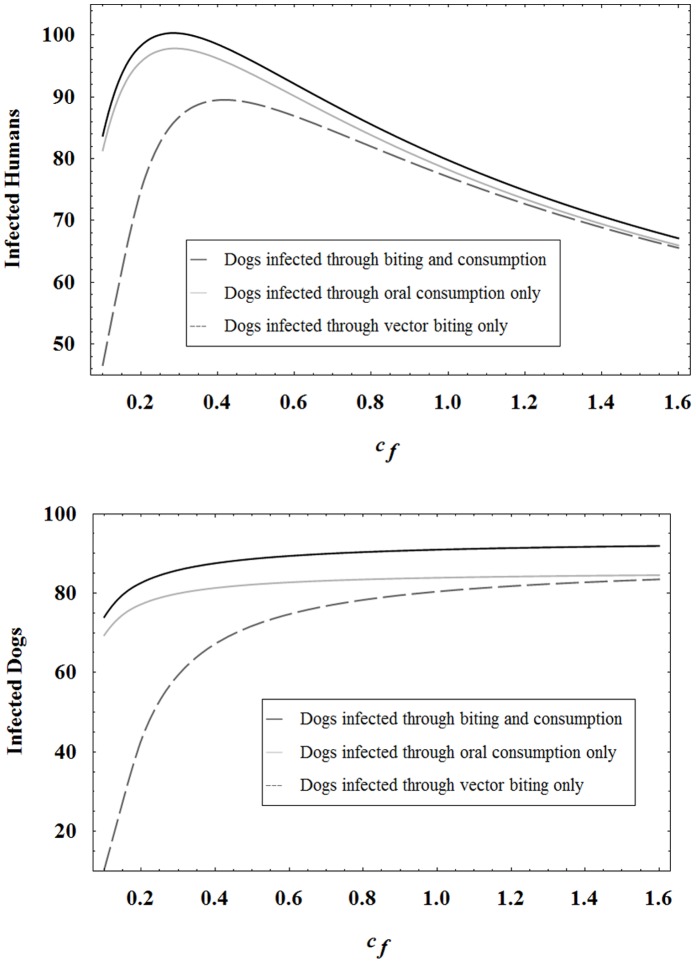
Infected humans and infected dog populations at year 30 in different scenarios. The infected human and infected dog levels after 30 years in different scenarios where dogs can be infected through vector biting only, oral consumption only, or both biting and consumption. Here 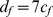 and all other parameters are set to the baseline values.

Past work has shown that dogs play an important role in the infection cycle. The new model, and particularly the inclusion of oral transmission, demonstrates an even more severe, negative impact of the dogs. In fact, Figures 7 and 8 reveal that high levels of human infection occur in both of the following cases: a high level of dog oral transmission coupled with a low vector biting preference for dogs and chickens; a low level of dog oral transmission coupled with a high vector preference for dogs and chickens. The key observation is that even if vector preference for dogs is low, dogs still become sufficiently infected through oral transmission to drive infection in vectors and humans. Furthermore, for low levels of 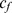 and 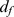, dog infection is actually caused more by oral consumption of vectors than by vector biting. See Figure 9. The figure further reveals that human infection remains high at low 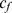 and 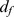 values even when dogs cannot be infected through biting.

### Sensitivity to Consumption

**Figure 10:**
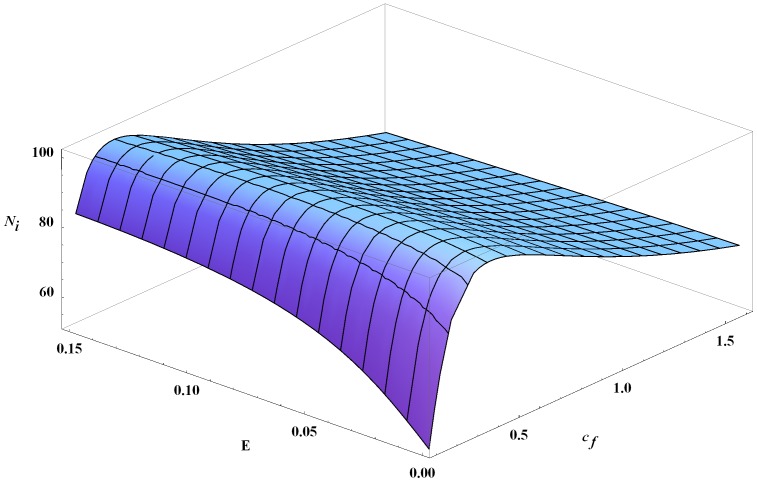
Infected humans at year 30 as a function of 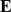 and 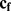. The number of infected humans at year 30 as a function of 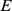 and 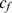, where 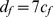 and all other parameters are set to the baseline values.

As shown above and in Figure 10, the model is sensitive to the value of 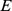, but only at low values of 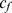 and 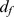. For example, when 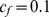 and 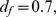 and 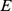 ranges from 0 to 0.15, the number of infected humans changes by about 30. However, the model is not very sensitive to 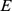 for larger values of 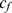 and 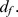 In fact, for any value of 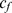 greater than the baseline value of 0.35, the number of infected humans at year 30 changes by at most 4 as 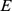 varies in the same range (0 to 0.15).

### Varying Dog and Chicken Levels

**Figure 11:**
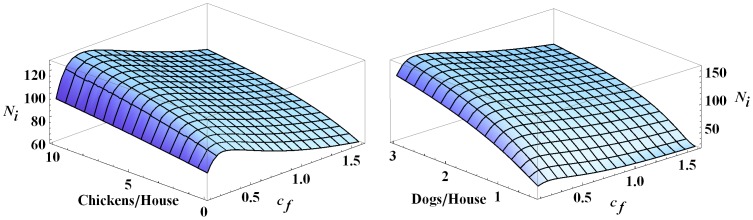
Infected humans at year 30 with different numbers of chickens and dogs. The figure shows the effects of changing the number of chickens and dogs on the number of infected humans in the village at year 30. Note that 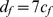 and all other parameters are set to their baseline values.

We now investigate the effects of changing the number of chickens and dogs in the village and their preference factors, while setting all other parameters to their baseline values. For fixed 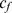, the number of infected humans increases as the number of chickens increases, Figure 11. Although chickens are not infectious, a higher chicken population means a higher blood supply is available to the vectors, resulting in higher vector prevalence. This leads to more bites on dogs and humans and increased infected populations.

Similarly, for a fixed value of 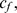 the number of infected humans increases as the number of dogs increases, Figure 11. The effect on 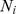 of increasing the number of dogs is more dramatic than the effect of increasing the number of chickens because dogs are infectious. As can be seen in both cases, the number of infected humans initially increases with increasing 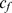 before decreasing once 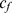 is beyond about 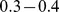.

### Larger Blood Supply Cases

**Figure 12:**
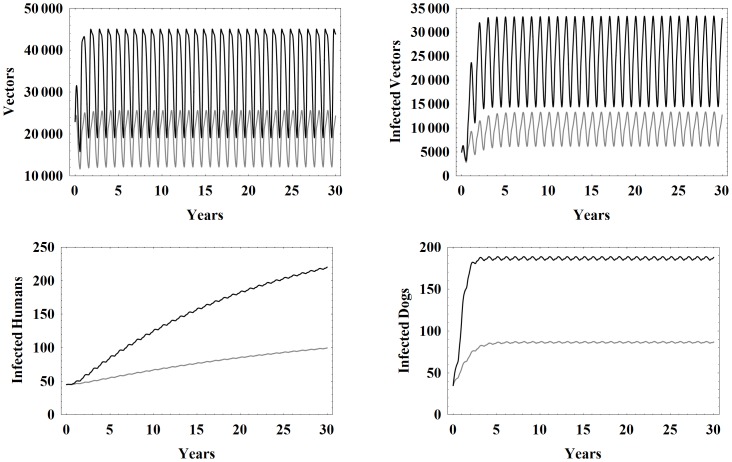
Populations with different blood supplies. Simulation results of the model with baseline parameters (gray) and with a higher blood supply (black). The higher blood supply case has 5 humans, 2 dogs, and 18 chickens per house in the village with all parameters set to the baseline values.

We now consider a more realistic village, see [36], where each house in the village has 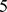 humans, 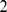 dogs, and 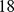 chickens. All other parameters are taken to be the baseline values. The primary effect of the increased populations is a larger blood supply available to the vectors. Correspondingly, the total number of vectors is significantly higher than in the baseline case. See Figure 12. All of the infected populations are significantly higher and a greater percentage of all populations become infected.

## Discussion

This work presents a new model with seasonally dependent coefficients for the domestic transmission of Chagas disease, building upon the work in [21]. The model includes transmission through vector biting along with the new infection routes of congenital transmission in humans and domestic mammals as well as oral transmission in domestic mammals through consumption of infected vectors. Simulations indicate that oral transmission plays an important role in the infection cycle while the effect of congenital transmission is more limited.

The inclusion of congenital transmission in humans directly leads to more human infections. However, because both the birth rate and the probability of the infection passing from mother to child are relatively low, vertical transmission in humans leads to only a few new infections over 30 years in a village of 400. We note that the choice to have a constant human population limits the model's flexibility in choosing the birth rate and thus it is perhaps artificially low. Clearly, the effects of congenital transmission in humans would be more severe in villages with higher birth rates. On the other hand, since dogs have a relatively higher birth rate than humans, congenital transmission in dogs might be expected to substantially influence the number of infected dogs and indirectly increase the number of infected humans. However, the dogs become so easily infected by other transmission routes that the addition of congenital transmission to the model does not significantly affect dog infections. In particular, simulations show that the infected dog population quickly stabilizes at a high level of infection (usually around 60%–90% infected). The inclusion of congenital transmission does slightly increase the peak dog infection level, but not enough to substantially affect human infection.

The effects of oral transmission in dogs are more dramatic and complex than that of congenital transmission. Furthermore, the significance of the oral transmission is strongly tied to the vector biting preference numbers. For high values of 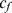 and 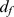, most of the dogs become infected through biting without oral transmission, though increasing the dog's consumption rate does moderately increase the number of infected dogs (Figure 8). However, in this case, the increased consumption causes a decline in the infected vector population, and correspondingly, in the infected human population. These declines are small though, which demonstrates that vector biting drives infection when the vectors strongly prefer to feed on dogs.

Alternatively, oral transmission is the driving force behind the infection cycle when 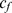 and 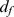 are low (Figure 7). In this case, vector biting alone leads to only about 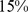 of the dogs being infected after 30 years. However, adding oral transmission dramatically increases all of the infected populations and the level of infection is very sensitive to the dog's maximal consumption rate, 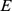. As noted in Results, the number of human infections in a representative village of 400 increases by about 30 infections over 30 years of simulation as 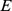 is increased from 0 to the baseline value of one per week. This means that with significant oral transmission in dogs, human infection will remain high even if the vectors have a low preference for biting the dogs. Since the probability of transmission from dogs to vectors is significantly higher than the probability of transmission from humans to vectors, see Table 1, we know that the dogs are primarily responsible for infecting the vectors. In turn, the size of the infected vector population directly drives the number of human infections. So, when 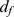 is low, oral transmission is the key route of dog infection, whereas biting is more important when 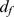 is high. In either case, our simulations show that the level of dog infection remains high, resulting in a substantial number of human infections. This result is noteworthy because it suggests that the disease will persist at high levels even if measures are taken to deter the vectors from biting the dogs, e.g. using insecticide collars.

It is well-known and widely reported that domestic mammals are a major player in the infection cycle and the main reservoir of the parasite. Our work strengthens these conclusions and further demonstrates the need to remove mammals (dogs, cats, etc.) from the domestic settings. This is not a new control recommendation, but our simulations, and particularly the inclusion of oral transmission, show the fundamental, negative role the mammals play in causing human infections over a wide range of vector biting preferences. In fact, our simulations show that the infections persist endemically even with a small number of dogs (0.2 dogs/home). This is consistent with [34], where it was found that a 100% effective control method on at least 88% of the dogs would be needed to achieve a basic reproductive number smaller than 1.

In our model, the infection dies out with no dogs. And even if a total removal of domestic mammals is infeasible, reducing their numbers will likely lead to fewer human infections. As shown in Results, the number of infected humans only increases with the number of dogs in our model. This contrasts with the model in [24], where it was found that human infection declines when each household has more than two dogs, allowing the dogs to sufficiently divert vectors away from the humans. In our model, more dogs means a higher blood supply available to the vectors, and correspondingly, more vectors and higher infected populations. However, we note that we do see a similar decline in humans infections as the vector biting preference for dogs increases beyond about 2.5 human factors. See Figure 4.

We note that this work uses a predation term to model the oral transmission in dogs, and this term may not appropriately account for other likely routes of oral transmission such as licking of feces-contaminated fur and ingesting feces-contaminated food or water.

A weakness of this work is that the parameters are coming from different studies. However, data from the same studies do not currently exist. Although the simulation results are highly sensitive to the vector biting preferences for dogs and chickens, which are largely unknown, the primary control implication–eliminating domestic mammals–is independent of these parameters. That is, domestic mammals should be removed from the homes even if vector preference for them is low. Furthermore, due to recent work in [33], we have drastically changed the relationship between 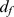 and 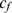 as compared to our previous work [21], [22]. Yet, the overall dynamics are very similar to our previous work and dogs remain the driving force of the infection cycle.

The dynamics of Chagas disease are indeed complex, so in addition to validation of the model, there are several open issues that are of interest for further study. First, we point out that human infection levels might be higher than in the simulations, because we did not include blood transfusions or oral transmission in humans, though recent outbreaks (e.g. feces in juice) indicate that the latter may be a significant source of human infection. Additionally, the effects of wild vectors and the disease in the wildlife were not investigated. We also did not directly consider vector mortality due to consumption by domestic non-mammals. Finally, the dynamics for the total human and domestic animal populations could be studied, which would allow for investigation of immigration and contact between neighboring villages.
